# Hepatocellular Carcinoma Automatic Diagnosis within CEUS and B-Mode Ultrasound Images Using Advanced Machine Learning Methods

**DOI:** 10.3390/s21062202

**Published:** 2021-03-21

**Authors:** Delia Mitrea, Radu Badea, Paulina Mitrea, Stelian Brad, Sergiu Nedevschi

**Affiliations:** 1 Department of Computer Science, Faculty of Automation and Computer Science, Technical University of Cluj-Napoca, Baritiu Street, No. 26-28, 400027 Cluj-Napoca, Romania; Delia.Mitrea@cs.utcluj.ro (D.M.); paulina.mitrea@cs.utcluj.ro (P.M.); sergiu.nedevschi@cs.utcluj.ro (S.N.); 2 Medical Imaging Department, Iuliu Hatieganu University of Medicine and Pharmacy, Cluj-Napoca, Babes Street, No. 8, 400012 Cluj-Napoca, Romania; rbadea@umfcluj.ro; 3 Regional Institute of Gastroenterology and Hepatology, Iuliu Hatieganu University of Medicine and Pharmacy, Cluj-Napoca, 19-21 Croitorilor Street, 400162 Cluj-Napoca, Romania; 4 Department of Design Engineering and Robotics, Faculty of Machine Building, Technical University of Cluj-Napoca, Muncii Boulevard, No. 103-105, 400641 Cluj-Napoca, Romania

**Keywords:** hepatocellular carcinoma (HCC), contrast-enhanced ultrasound (CEUS) images, multimodal combined CNN classifiers, feature level fusion, classifier level fusion, decision level fusion

## Abstract

Hepatocellular Carcinoma (HCC) is the most common malignant liver tumor, being present in 70% of liver cancer cases. It usually evolves on the top of the cirrhotic parenchyma. The most reliable method for HCC diagnosis is the needle biopsy, which is an invasive, dangerous method. In our research, specific techniques for non-invasive, computerized HCC diagnosis are developed, by exploiting the information from ultrasound images. In this work, the possibility of performing the automatic diagnosis of HCC within B-mode ultrasound and Contrast-Enhanced Ultrasound (CEUS) images, using advanced machine learning methods based on Convolutional Neural Networks (CNN), was assessed. The recognition performance was evaluated separately on B-mode ultrasound images and on CEUS images, respectively, as well as on combined B-mode ultrasound and CEUS images. For this purpose, we considered the possibility of combining the input images directly, performing feature level fusion, then providing the resulted data at the entrances of representative CNN classifiers. In addition, several multimodal combined classifiers were experimented, resulted by the fusion, at classifier, respectively, at the decision levels of two different branches based on the same CNN architecture, as well as on different CNN architectures. Various combination methods, and also the dimensionality reduction method of Kernel Principal Component Analysis (KPCA), were involved in this process. These results were compared with those obtained on the same dataset, when employing advanced texture analysis techniques in conjunction with conventional classification methods and also with equivalent state-of-the-art approaches. An accuracy above 97% was achieved when our new methodology was applied.

## 1. Introduction

Hepatocellular Carcinoma (HCC) is the most common malignant liver tumor, which appears in 70% of liver cancer cases. Nearing the top of the most frequent tumors worldwide, HCC is placed at fifth position. It is also situated at the fourth position at the top of cancer-related deaths around the world [1]. HCC usually evolves from cirrhosis, after a liver parenchyma restructuring phase when dysplastic nodules result, which can transform into HCC. The golden standard for HCC diagnosis is, nowadays, the needle biopsy, but this is an invasive, dangerous technique, as it can lead to the spread of the tumor inside the human body and also to infections [2]. Thus, alternative, safer techniques based on advanced computerized processing and artificial vision are due. Ultrasonography is a medical examination method that is cheap, non-invasive, non-irradiating and, thus, repeatable, suitable for patient-disease monitoring. Other medical image-based examination methods, such as Computer Tomography (CT), Magnetic Resonance Imaging (MRI) and endoscopy are irradiating and/or expensive. Contrast-Enhanced Ultrasound (CEUS) imaging is an improved ultrasound-based technology, assuming the injection into the blood of a specific contrast agent, consisting of gas filled microbubbles. The contrast agent spreads through the human body, emphasizing the vessel structure in the region of interest [3]. This technology leads to the highlighting of both large vessel flows, as well as of the microcirculation, being firstly implemented for hepatic tumor pathology, for abdominal emergencies and in order to recognize various tumor types [4]. The microbubbles of the contrast agent produce harmonic echoes, which are detected by the transducer. This behavior is significantly different from that of the usual ultrasound waves reflected by the tissues. The ultrasound devices employ ultrasound emissions to cancel the tissue signals and to emphasize those of the microbubbles. The CEUS technology reported a superior sensitivity, compared to that of CT or MRI with a gadolinium- or iodinated-based agent [5].

**Figure 1:**
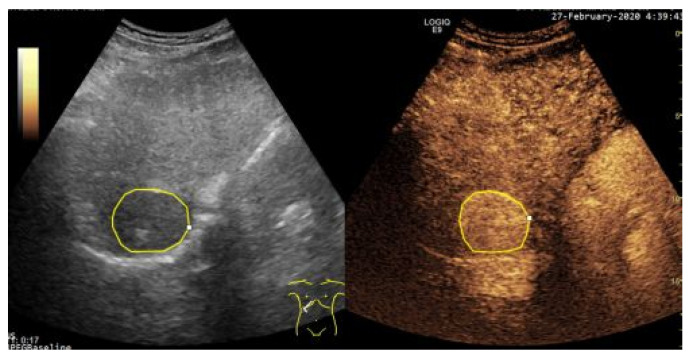
Example of a Hepatocellular Carcinoma (HCC) tumor within B-mode Ultrasound (**left**) and Contrast-Enhanced Ultrasound (CEUS) images (**right**), delineated by an experienced radiologist.

Within B-mode ultrasound images, focal, encephaloid HCC appears, in more advanced evolution phases, as a well-defined region, of 3–5 cm in size, being hyperechogenic and often heterogeneous, due to the interleaving of fatty cells, necrosis, fibrosis and active growth tissue [2]. In CEUS images, HCC appears more highlighted, due to the dense and complex vessel structure that is specific to the malignant tumors [3]. The HCC tumors are usually hyper-enhanced during the arterial phase, showing washout during the portal venous and delayed phases [5]. An eloquent example of HCC tumor, as it appears in a pair of B-mode Ultrasound and CEUS images, is depicted within Figure 1. However, in many cases, within both B-mode ultrasound images and CEUS images, HCC is hardly distinguishable from the cirrhotic parenchyma on which it evolves, so advanced computerized methods are due, in order to overpass the limitations of the human eye, in a non-invasive manner.

In this context, adopting appropriate methods for noise removal is of great importance. The noise and the artifacts, due mainly to the acquisition process, might lead to uncertainty and imprecision, causing undesired phenomena within the medical images, such as the partial volume effect [6]. Concerning the ultrasound images, the most frequent type of noise is that of speckle, affecting all the types of ultrasound images [7]. In addition, the quality of the CEUS images is lower in the deeper regions, presenting a low Signal-to-Noise (SNR) ratio, due to the bubble disruption phenomenon [8]. Low-pass filters, such as the median filter or the arithmetic mean filter [9], the transform-based methods [7,8], as well as the fusion of multiple image modalities [6] were successfully implemented for reducing noises within medical images. Contrast enhancement through specific computerized methods is of great importance also, in both real-world and medical images, in order to highlight the objects of interest, the anatomical or pathological structures, to reduce uncertainty or imprecision. Such relevant methods are described in [10], respectively, in [11]. In [10], the authors present their method for adaptive contrast enhancement in gray level images. An S-shaped fuzzy Membership Function (MF) was defined for this purpose. Then, a set of fuzzy patches were extracted, ascending order statistics being computed on these patches. These statistics were considered as points in a 4-D fuzzy unit hyper-cube, which finally led to contrast enhancement, based on the distances between the previously mentioned points, respectively those of maximum darkness and maximum brightness. A new approach regarding the histogram equalization technique was presented in [11]. Histogram division was performed by automatically estimating the number of sub-histograms, based on the number of peaks of the original histogram. Then, corresponding clusters were defined by grouping the pixels based on their intensity levels with the aid of a fuzzy clustering algorithm. Finally, the histogram equalization method was applied for each cluster separately. The obtained results demonstrated an obvious increase concerning the image clarity. In addition, a more natural appearance of the elements within the images of the experimental set was gained.

Many approaches exist, performing the recognition, within medical images, of various important affections, including tumors, by employing specific image analysis and machine learning techniques [12,13,14]. Initially, the texture-based methods in combination with classifiers have been widely exploited [15,16,17,18]. Specific methods, such as the Gray Level Co-occurrence Matrix (GLCM) [17], the Run-Length Matrix [18], the Wavelet [13] and the Gabor transforms [16], in combination with powerful machine learning techniques, such as Support Vector Machines (SVM) [13], Artificial Neural Networks (ANN), Fisher Linear Discriminants (FLD) [19] or the Bayesian classifiers [16] have been extensively implemented in this context. Recently, the deep learning techniques, such as the Stacked Denoising Autoencoders (SAE) [20], the Deep Belief Networks (DBN), the Recurrent Neural Networks (RNN), respectively, the CNN-based classifiers, were successfully employed for automatic diagnosis within medical images [21,22]. They demonstrated their value in other fields, as well, such as bioinformatics [23,24], object detection and recognition [25], semantic segmentation of images [26]. CNN began to be utilized on a large scale when powerful computational resources, such as the parallel units or the Graphical Processing Units (GPU), appeared—their value being emphasized during the ImageNet competition in 2012. They were also included in the top 10 most important discoveries in [27]. This technology demonstrated its superiority in many situations, such as fatty liver recognition within ultrasound images [14], fibrosis grade detection for patients affected by B-type hepatitis from 2D shear wave elastographic images [28], breast tumor recognition within ultrasound images [29], liver lesion recognition [30], liver tumor recognition [31] and segmentation [32], pulmonary nodule detection from CT images [33]. Standard CNN architectures, as well as original, deep CNNs, were employed for these objectives.

The CNN-based methods also demonstrated their value in the field of bioinformatics. In [23], the authors described their methodology, aimed at classifying DNA promoters through the interpretation of the DNA sequences. They employed deep learning and appropriate text processing techniques for this purpose. The DNA promoters represent short regions inside DNA where specific genetic processes occur, being the main cause of many important affections, as diabetes, cancer, or Huntington’s disease. In their innovative approach [23], the authors modeled the DNA sequences as a combination of continuous FastText N-grams, which were provided at the input of an 1D CNN with an original architecture. After the experiments, an accuracy of 85.41% resulted when distinguishing between promoter and non-promoter, while in the case when differentiating between strong and weak promoters, the accuracy was 69.4%. This performance was superior to that of other relevant state-of-the-art methods. Within another representative approach for this domain, 2D CNN architectures were employed for predicting the Flavin Mono-Nucleotide (FMN) binding sites [24]. The FMN co-factors are responsible for carrying and transferring electrons during the cellular respiration, being able to provide information about diseases, also upon drug targets. The Position Specific Scoring Matrix (PSSM) data was provided at the entrances of a 2D CNN with an original architecture. This architecture consisted of zero padding 2D layers, convolution 2D layers and max pooling 2D layers, respectively, of a flattened layer, two fully connected layers separated by a dropout layer and a *softmax* layer, within the output section. After performing 5-fold cross validation, an accuracy of 98.2%, a sensitivity of 83.7%, a specificity of 99.2% and a Matthews correlation coefficient of 0.85 resulted. This performance overpassed the previous state-of-the-art results.

Both conventional methods and deep learning techniques were also applied for performing tumor recognition and segmentation within contrast-enhanced medical images [34,35,36,37], respectively, within combinations of different medical image modalities [26,38], the CNN techniques having an important role in this context. The most relevant approaches in this field are described below.

### 1.1. Automatic Diagnosis Approaches within Contrast-Enhanced Medical Images

In [34], the authors presented a two-stage multi-view learning framework for performing the automatic diagnosis of the liver tumors from CEUS images. Three typical CEUS images, corresponding to the arterial phase, portal venous phase and late phase were adopted. Firstly, the Deep Canonical Correlation Analysis (DCCA) was applied upon the three image groups, yielding six-view features. During the second stage, the multi-view features were provided at the entrances of a Multiple Kernel Learning (MKL) classifier, for the automatic recognition of the liver tumors. After applying the above-described methodology, a sensitivity of 93.56%, a specificity of 86.89% and an AUC of 95.3% resulted. The study described in [39] highlighted the implementation of an automatic classification method within CEUS images, which employed the contrast agent Sonazoid, in order to discover the presence of the Focal Liver Lesions (FLLs), such as HCC, benign and metastatic liver tumors. The authors determined spatial, as well as temporal features, during the arterial phase, portal phase, post-vascular phase, respectively, within max-hold images. These data were provided to an SVM classifier, for supervised classification. The images corresponding to 98 subjects were included in the experimental dataset, yielding, in the case of malignant/benign structure differentiation, an accuracy of 91.8%, a sensitivity 94.0% and a specificity of 87.1%. For HCC, benign and metastatic tumor classification, the accuracy was 85.7%, the sensitivity was 84.4% and the specificity was 87.7%. Another approach that performed the recognition of the liver lesions based on dynamic CEUS images was presented in [40]. The video sequences containing the CEUS frames were processed by a 3D CNN, which yielded spatial and temporal features. The corresponding framework was trained using a specific dataset. This system achieved an average accuracy of 93.1%, when the 10-fold cross-validation strategy was employed. In [41], the authors assessed a radiomics methodology based on deep learning techniques to derive features in order to evaluate the Progression-Free Survival (PFS), as well as the Surgical Resection (SR) of the Radiofrequency Ablation (RFA) procedure. Another objective was that of optimizing the selection of patients with incipient HCC, supposed to undergo specific treatments. The study included the CEUS images of 419 patients, which were examined one week before the RFA or SR procedures. Then, RFA and SR nomograms were built based on Radiomics signatures and relevant clinical variables. This methodology led to a satisfying accuracy concerning the targeted discrimination and predictions—the C-index being 0.736 for RFA, respectively, 0.741 for SR. The CEUS technology was also involved in the detection of the colorectal liver metastases, as illustrated in [42]. According to the clinical studies, the contrast agent led to the discovery of a more increased number of metastases, with improved sensitivity and specificity, in comparison with the B-mode ultrasound technology. The existing studies highlighted that CEUS had a considerably increased sensitivity, of 80–90%, regarding the detection of the liver metastases, comparable with the CT technology. In addition, some of the reports demonstrated that CEUS manifested a specific sensitivity to metastases smaller than 10 mm, improving the corresponding sensitivity by approximately 50%, compared with the classical US. A method that performed automatic tumor segmentation within a dynamic sequence of CEUS images was described in [36]. A new CNN architecture, named CEUS-Net, was defined and assessed. This network was derived from the well-known U-Net architecture, infused with newly designed feature-re-weighted dense blocks, for selecting relevant information, an attention mechanism being implemented. A multichannel convolutional module made the learning of spatial-temporal features possible. The novel CEUS-Net architecture was experimented in order to segment breast and thyroid lesions. The corresponding performance, measured through the dice index, was 0.84 in the case of the breast tumors, respectively, 0.78 for the thyroid tumors.

### 1.2. Automatic Recognition and Segmentation Using Multiple Image Modalities

In order to improve the automatic recognition process, the fused information derived from multiple data categories, in particular from multiple image modalities, was employed in both medical and real world images. Referring to medical image recognition employing the classical approach, in [38], the objective was the recognition of the liver tumors using the textural parameters resulted from classical, as well as from contrast-enhanced CT images. The texture-based attributes resulted from the classical CT images were concatenated with those obtained from the contrast-enhanced CT images. After relevant attribute selection, a C4.5 classification method was implemented, yielding a classification accuracy over 90%. Regarding the deep learning techniques applied in the domain of medical image recognition when considering multiple image modalities, in [43], the authors proposed a newly defined Temporal Sequence Dual-Branch Network (TSDBN) that used both B-mode ultrasound and CEUS images, in order to diagnose the breast tumors. A Gram matrix was employed for performing temporal modeling of the CEUS image sequence. Then, a Temporal Sequence Regression Mechanism (TSRM) was involved. This mechanism represented a new method for extracting powerful features from the CEUS frames, based on the previously defined matrix. Regarding the network structure, two separate branches based on different ResNet versions were considered for feature extraction, within B-mode ultrasound and CEUS images. The TSRM method was involved in order to obtain a temporal sequence relationship between the frames, also in order to design a Shuffle Temporal Sequence Mechanism (STSM) for the temporal sequences. According to the experimental results, the proposed methodology led to an accuracy improvement close to 4%, with respect to the previously existing state-of-the-art approaches. Another relevant approach was presented in [44]. The authors combined histological and immunohistochemical image data (of type PR, ER, Her2 and Ki-67) for breast cancer diagnosis. Three basic CNN architectures, VGGNet, ResNet and InceptionV3 were considered for combination and two transfer learning strategies were adopted. The first strategy assumed to use the pre-trained DCNN architectures for feature extraction, then the features corresponding to the five image types were concatenated. Thereafter, the Principal Component Analysis (PCA) method was used for dimensionality reduction, followed by a Linear Discriminant Analysis (LDA) classifier. The second strategy assumed to provide the images of the five stain types to a multi-input DCNN, the corresponding branches being previously trained with appropriate data. Thus, only the weights of the last layers were adjusted, by training them with the specific data. The first strategy performed better, a sensitivity above 89% being attained. In [26], the authors aimed to achieve the segmentation of the sarcoma malignant tumor by combining MRI, CT and Positron Emission Tomography (PET) images. The contour of the images in the training set was manually delineated by the medical specialists. Three approaches are compared: (a.) firstly, the fusion of the features individually determined on each image type, containing multi-modal intrinsic representations of the image data was performed; then, the result of the fusion process was provided as input to a CNN network, designed by the authors (feature level fusion); (b.) only the parts which performed convolution were separated within the network architecture, so that the images of each category were individually provided and processed in parallel, by identical convolution units; then, the results were processed by a single fully connected network, which determined the class of belongings (classifier level fusion); (c.) the corresponding images of various types were provided to different CNNs, the final result being established through a majority voting procedure (decision level fusion). Finally, the feature and classifier level fusion approaches provided the best results, the segmentation accuracy being around 95%. The combination of multi-modal PET images was exploited in [6]. The aim was to improve the segmentation process, in particular concerning tumor localisation, in conditions of uncertainty and imprecision, inherent to the nature of the images. For this purpose, the fusion of multi-tracer PET functional medical images, combined with elements of the belief function theory, was employed. The neighboring information derived from mono-modal images, as well as a priori contextual knowledge, regarding the spatial resolution of the acquisition system, were also considered. Appropriate combination rules were then applied for information fusion. The proposed methodology provided good results on both simulated images with various SNR ratios, as well as on medical images.

Concerning the combination of multiple image types in order to perform automatic recognition within real-world images, relevant approaches that refer to action recognition [45], object recognition [46], and complex geomodel recognition, respectively, exist. In [45], the authors proposed a methodology based on CNN techniques for combining spatial and temporal information received from multiple sources. The final purpose was that of performing action recognition. An additional feature, based on optical flow, was also added to the original dataset. Different CNN branches were employed for each data source and the feature maps representing the results of these CNN branches were combined by a multiplicative fusion method. The aim was to amplify or suppress the feature activations, based on their agreement. The obtained results were comparable with the state-of-the-art results. In order to accurately perform object recognition, in [46], the authors employed CNN-based techniques for exploiting both color and depth information (RGB-D data). A two-stage cascaded network was implemented for this purpose. This network was designed in order to perform both PCA and canonical correlation analyses. Within the first layer, the network assimilated PCA based filters for depth and RGB. The second layer was destined to canonical correlation analysis filter jointly learning for the two modalities, RGB and depth. An accuracy of 91% resulted after experimenting this methodology. A study concerning the parametrization of the complex 3D geomodels through a deep learning methodology was described in [47]. A 3D CNN-PCA algorithm was designed for this purpose, which employed a CNN as a postprocessor for low level PCA representation. The CNN-PCA method yielded geological features that were consistent with the reference models. This algorithm was also applied for history matching within a bimodal channelized system.

As it results from the above-described methods, the contrast-enhanced medical images demonstrated very good results concerning the automatic diagnosis of both liver tumors and other tumor types. In addition, combining multiple image modalities generally led to significant improvements of the recognition and segmentation accuracy in both medical and real-world images. However, no relevant approach exists that employs both B-mode ultrasound and CEUS images for performing the automatic recognition of HCC based on CNN architectures. We performed this in our current research, by combining the two types of information at different levels, also involving various combination functions and the KPCA dimensionality reduction method for this purpose. Within our preliminary research presented in [48], we firstly analyzed the accuracy improvement due to the combination between CEUS and B-mode ultrasound images, by employing conventional approaches, respectively, the SAE deep learning classifier. The conventional approaches consisted of providing the relevant textural features and the KPCA result, respectively, corresponding to B-mode ultrasound images, CEUS images, and to the combination between B-mode ultrasound and CEUS images at the entrances of powerful traditional classifiers. As feature selection methods, we employed Correlation-based Feature Selection (CFS) and Information Gain Attribute Evaluation (IGA). Regarding the traditional classifiers, we adopted the SVM technique, the Multilayer Perceptron (MLP), and AdaBoost combined with decision trees. On the other hand, the SAE deep learning classifier received as inputs the relevant textural feature sets, or the pixel intensity values of the original images. When assessing the value of the combined B-mode ultrasound and CEUS information, feature level fusion, classifier level fusion and decision level fusion were considered, regarding this classifier. The best performance was achieved for the SAE classifier, when employing feature level fusion. Other relevant papers, representative for our research, are [9,49,50], where we performed the automatic recognition of the HCC tumors, within B-mode ultrasound images, on various datasets, by employing advanced texture analysis methods combined with conventional classifiers, both, respectively, CNN techniques.

### 1.3. Contributions

As previously stated, no relevant approach exists that performs HCC recognition within combined B-mode ultrasound and CEUS images, through CNN-based techniques. In addition, none of the existing approaches compare the HCC recognition performance based on B-mode ultrasound images, with that due to CEUS images, by taking into account CNN techniques. Within the current research work, the objective was that of assessing the role of the CEUS images, employed individually, or in combination with B-mode ultrasound images, concerning the HCC automatic diagnosis, by using CNN techniques. Thus, the contributions of the current research are the following: (a.) Compare the HCC recognition performance achieved by using B-mode ultrasound images, CEUS images, and combined B-mode ultrasound and CEUS images, respectively, when employing CNN architectures. (b.) Assess various CNN architectures with different parameters in order to get the best performance in the above-mentioned situations. (c.) Experiment and evaluate multiple methods in order to combine B-mode ultrasound images with CEUS images, such as feature level fusion, classifier level fusion and decision level fusion; assess various methods of feature level fusion (arithmetic mean, weighted mean and multiplication of the input images); concerning the classifier level fusion, assess the role of various combination modalities (concatenation, arithmetic and weighted mean, multiplication, KPCA with different kernels), in order to combine the outputs of the convolutional parts, which corresponded to CNN structures having the same architecture, as well as different architectures, at various levels. Various types of multimodal combined classifiers resulted in this manner, which were finally compared. (d.) Compare the role of the CNN-based techniques with conventional approaches, based on texture analysis and traditional classifiers, in each experimental case.

## 2. Materials and Methods

### 2.1. Background

In the context of the current research, we aim to assess the role of the CNN architectures, respectively of their combinations, regarding the automatic recognition of the HCC tumors within combined B-mode ultrasound and CEUS images. The role of various combination methods, including the KPCA dimensionality reduction technique, is assessed in this context. Thus, within the next paragraphs, we will synthesize the theoretical foundations corresponding to these methods.

#### 2.1.1. Convolutional Neural Networks (CNNs)

As stated before, the CNN techniques reached, during the last decade, the top of the most valuable methods for image classification. They are also integrated in the deep learning techniques category, as they represent a class of feed-forward artificial neural networks, having more than three hidden layers. They simultaneously perform dimensionality reduction, image analysis and recognition. CNNs are different from other types of neural networks, as they consist of combining multiple MLP structures, organized in convolutional layers, employed in order to compress the data in recognized patterns (models) [51]. A CNN induces a local connectivity model between the neurons belonging to neighbouring layers. Thus, the entrances of the hidden units of the m-th layer are provided by a subset of units from the layer m-1—these units having spatially contiguous receptive fields. Within the CNN, each convolution filter is extended upon the whole visual field. The replicated units (the convolutional filters) have, as principal parameters, the Weight (W) vector and the distortions (Bias (B)). At each level, the input image is convolved with a set of K filters, having the weight vector $W=\{W_{1},W_{2},\ldots ,W_{K}\}$, respectively, the biases $B=\{b_{1},\ldots ,b_{K}\}$, each of them generating a single feature map $X_{k}$, k∈{1,…,K}. The corresponding features are subjected to a non-linear transform, this process being repeated on each convolutional layer, *m*, the mathematical formula being presented in (1) [21]:

(1) $$X_{k}^{m}=\sigma \left(W_{k}^{m-1}X_{k}^{m-1}+b_{k}^{m-1}\right)$$

In (1), $W_{k}^{m},k\in \left\{1,\ldots ,k\right\}$ is the vector of weights, which connects the pixels from the layer *m*, having the coordinates (i,j), with the corresponding pixels from the layer m−1, having the same coordinates. Relevant examples of the σ() transform are the *hyperbolic tangent(tanh)*, respectively the *sigmoid* function [21]. CNN also incorporates pooling layers, where the feature values are aggregated through a permutation invariant function, which usually computes the average, or extracts the maximum value. These operations target the computational complexity reduction at superior levels within the CNN, also inducing translation invariance. Within a CNN, the *Rectified Linear Unit (ReLU)* layers can be met as well, which apply unsaturated activation functions of low cost, finally leading to the enhancement of the nonlinear properties of the CNN. The last part of the CNN is represented by fully connected layers, which perform supervised classification, at the end a class probability distribution being generated, with the aid of a *softmax* function.

Specific CNN architectures have been elaborated during the last decade, in order to achieve the best performances regarding the image recognition task. Firstly, the LeNet and AlexNet CNN architectures were elaborated, the latter containing seven layers and a higher number of filters, 3 × 3, respectively 5 × 5 in size, classifying more object classes than its ancestor. In comparison with LeNet, AlexNet employs dropout instead of regularization, for reducing the danger of overtraining. SqueezeNet represents a smaller neural network, having fewer parameters, but usually the same performance as the AlexNet architectures. For increasing the efficiency, it employs fire modules consisting of a squeeze convolution layer with only 1 × 1 filters; the data being then directed to an expanded convolutional layer, having a mix of 1 × 1 and 3 × 3 filters [52]. The VGGNet architecture stands for a sequential network having 21 layers, the filter size of 3 × 3 being taken into account for all the convolutional operations, replacing also the former 5 × 5 convolutions, in order to increase the detection accuracy of the small local features. Maxpooling layers are always inserted between the convolutional layers. As it manages 140 milions of parameters, it has an increased computational complexity [53]. The GoogLeNet architecture is based on the so-called “inception” modules, which replace the sequential convolutions, performed in separate layers, with simultaneous convolutions—a fact that considerably increases the computational efficiency. The 1 × 1 convolutions are also employed in this context, for dimensionality reduction. The InceptionV3 and InceptionResNetV2 architectures belong to the same class [54]. In order to overcome the “vanishing gradient” problem, specific to deep neural networks, the ResNet architecture implements the so-called residual connections, assuming that, during the training process, some of the convolutional layers are being skipped, in order to avoid the gradient vanishing problem, which might appear for the deep networks [55]. Within the same family, the more complex DenseNet architecture employs an increased number of residual connections (each layer receives connections from all the preceding layers), the information flow between the CNN layers being maximized. DenseNet also implements feature map reuse, which considerably reduces the number of the network parameters, increasing the efficiency [56].

#### 2.1.2. Kernel Principal Component Analysis (KPCA)

PCA represents a dimensionality reduction technique that projects the initial data on a lower dimensional space where the main variation modes are highlighted. Mathematically speaking, PCA determines a linear mapping *M*, which maximizes the quantity $M^{T}cov\left(X\right)M$, where cov(X) is the covariance matrix of the dataset *X*. It was demonstrated that the linear mapping matrix *M* is formed by the first *d* eigenvectors which correspond to the highest *d* eigenvalues of the covariance matrix, built on the initial dataset, where the mean value for each feature vector was subtracted. KPCA, the generalized version of PCA, represents the transposition of PCA in a superior space, built using a kernel function. KPCA determines the eigenvectors of the kernel matrix *K*, which is built by applying the kernel function on the initial data. The mapping of the original data onto a lower dimensional dataspace is performed according to the Formula (2) [57]:

(2) $$y_{i}=\left\{\sum_{j=1}^{n}\alpha_{1}^{j}K\left(x_{i},x_{j}\right),\ldots ,\sum_{j=1}^{n}\alpha_{d}^{j}K\left(x_{i},x_{j}\right)\right\}$$

In (2), $\alpha_{k}$, k∈{1,…,d} are the eigenvectors of the covariance matrix in the initial space, $\alpha_{k}^{j}$ is the j-th element of $\alpha_{k}$, *K* is the kernel function, while *n* is the number of the instances in the dataset. The kernel function can have different forms, such as Gaussian, polynomial and linear, in the last case being equivalent to the classical PCA [57]. In our work, all the three versions of KPCA were experimented, the best obtained results being depicted.

### 2.2. The Proposed Solution

**Figure 2:**
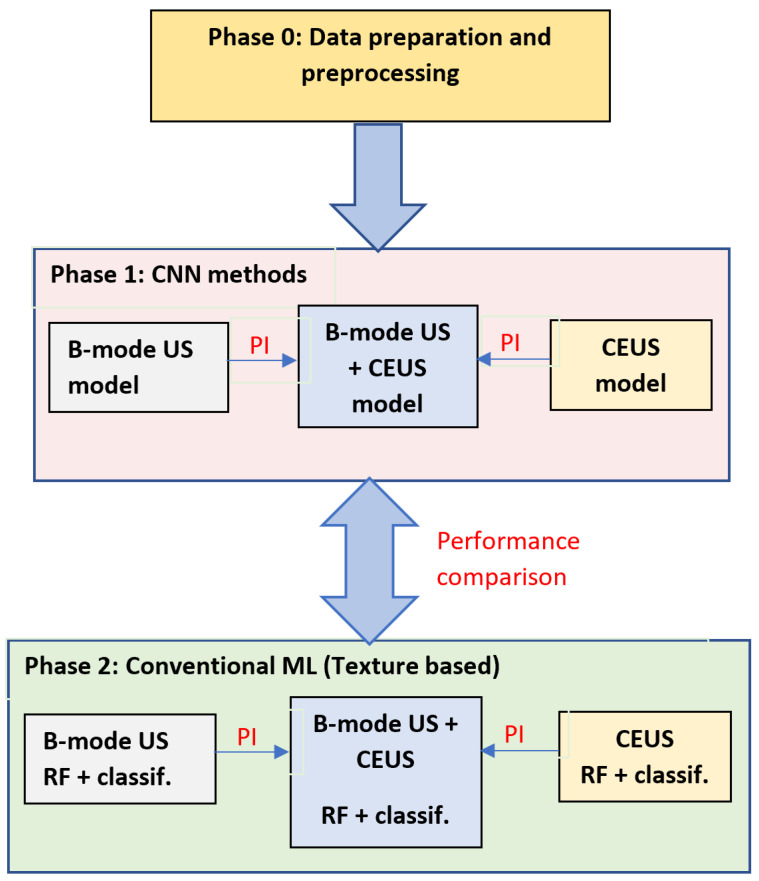
Methodology description. *Phase 0* corresponds to data preparation and preprocessing. *Phase 1* assumes the assessment of the CNN-based classifiers, on B-mode US and CEUS image separately, respectively on combined B-mode US and CEUS images, estimating the Performance Improvement (PI) for the last case. *Phase 2* consists of assessing a conventional Machine Learning (ML) approach, based on advanced texture analysis and traditional classifiers and comparing the corresponding performance with that obtained during Phase 1.

This research article aims to assess the role of the CEUS images, respectively of the combined B-mode ultrasound and CEUS images, concerning the automatic recognition of HCC by employing CNN-based technology and various fusion methods, such as the dimensionality reduction method of KPCA. The performance of the CNN techniques, resulted in this context, is also compared with a traditional approach, based on texture analysis and conventional powerful classifiers. A graphical schema of our methodology is depicted in Figure 2. As it results from Figure 2, during the first phase, various CNN architectures are employed, individually or appropriately combined, in order to assess the role of B-mode ultrasound images, of the CEUS images, respectively of the combination between the B-mode ultrasound and CEUS images in the automatic recognition of the HCC tumors. In order to combine the two image modalities, specific fusion methods were applied, at various levels: *feature level fusion*, assuming the combination of the original B-mode ultrasound and CEUS image data, the fused data being provided to a single CNN architecture; *classifier level fusion*, assuming the generation of multimodal combined classifiers, resulted through the fusion of two CNN branches, separately trained with B-mode ultrasound and CEUS data, before the fully connected or softmax layers; *decision level fusion*, implying to perform the arithmetic or weighted mean between the probabilistic results provided by two separate CNNs, trained with B-mode ultrasound, respectively with CEUS data. The classification performance due to the combination between the B-mode ultrasound and the CEUS images is compared with that separately obtained on each image type.

During the second phase, the performance resulted through the CNN technology was compared with that resulted by employing advanced texture analysis methods in conjunction with powerful classifiers.

#### 2.2.1. Data Preparation and Preprocessing

**(1.) Description of the dataset.** In order to perform the experimental assessments, according to the above-described methodology, the B-mode ultrasound, the CEUS images corresponding to 48 patients affected by HCC were considered. The HCC diagnostic of these patients was confirmed either through biopsy or CT examination. The corresponding images were acquired with the aid of a GE Logiq E9 XDclear 2.0 (General Electric, 5 Necco Street, Boston, MA 02210, USA) ultrasound machine, using identical settings: Frequency of 6 MHz, Gain of 58, Depth of 16 cm, Dynamic Range (DR) of 111. The Sonovue (Bracco^®^) agent was exploited during the CEUS examination. The CEUS images gathered during the arterial phase were included in our dataset, in order to get a better visibility of the HCC tumors. Pairs of CEUS and B-mode ultrasound images, which were acquired simultaneously, for each considered patient, were taken into account. These images were initially provided in DICOM format. Based on the DICOM videos, images in bitmap (.bmp) format, corresponding to separate frames, were extracted. The HCC tumors were manually delineated, within both CEUS and B-mode US images, by experienced radiologists, with the aid of the VGG Image Annotator (VIA) 3.0.6 application [58].

**(2.) Preparation and preprocessing steps.** Firstly, within the pairs of B-mode ultrasound and CEUS images, which had the HCC structures manually delineated by the radiologists, regions of interest of 51 × 51 pixels, for HCC and cirrhotic parenchyma where HCC had evolved were automatically extracted. This algorithm localized the center of each polygon and browsed a region of size 250 × 250 pixels around the center, using a sliding window algorithm. Within this region, pixels representing the centers of patches having 51 × 51 pixels in size were taken into account, with no intersection between the corresponding patches. If the size of the intersection with the polygon surrounding HCC was equal to 2601, the patch was integrated in the HCC class, while if the intersection was 0, the patch was integrated in the class of the cirrhotic parenchyma on which HCC had evolved, further denoted by *PAR*. The previously mentioned two classes were chosen for comparison, as the visual aspect of the HCC and PAR tissue in ultrasound images is very similar in many situations. The healthy liver tissue class was not included, as HCC usually evolves on cirrhhotic parenchyma. The data was uniformly split between these classes. Thereafter, the existing dataset was augmented by the 90, 180 and 270 rotation of the images, resulting 1500 patches/class. These data have been further augmented during training, through flip operations, respectively through translation operations.

#### 2.2.2. CNN Based Methods for HCC Recognition within Combined B-Mode Mode Ultrasound and CEUS Images

In order to perform HCC recognition within B-mode ultrasound and CEUS images, combined under various approaches, several representative CNN architectures, well known for their performance, were compared, such as:

(a) *SqueezeNet*, as a small and efficient CNN [52];
(b) *VGGNet*, as a classical, sequential CNN, well known for its performance [53];
(c) *GoogLeNet*, having an optimized CNN architecture, in comparison with its ancestors, due to the inception modules [59];
(d) *ResNet*, as a deep CNN, which implements the concept of residual connections [55];
(e) *An original version of GoogLeNet*, however, less complex than InceptionV3 and InceptionResNetV2, obtained by drawing residual connections in order to skip the last three inception modules, aiming to improve efficiency, while reducing the gradient vanishing danger; the residual connections were drawn from the output of each of these inception modules, to the network output, the addition operation being performed. In order to perform appropriate dimensionality reduction, respectively to equalize the feature vectors, which were inputs to the same addition unit, average pooling operations and 1 × 1 convolutions have been performed, achieving dimensionality reduction and the decrease in the number of parameters;
(f) *DenseNet*, as an improved version of ResNet, providing a maximized information flow through the network, but with less parameters.

These network architectures were chosen in order to include an exhaustive set of functional elements which were characteristic for CNNs: convolutions of various sizes, 1 × 1 convolutions, parallel convolutions, residual connections. These classifiers were experimented separately using each image type, as well as in order to fuse the two types of images, using the following approaches:

(1) Feature level fusion, the combination being performed directly among the data belonging to the B-mode ultrasound and to the CEUS images;
(2) Classifier level fusion, the combination being performed using the feature maps provided by two CNN structures, separately trained on B-mode ultrasound, respectively on CEUS data;
(3) Decision level fusion, assuming to compute the arithmetic or weighted means between the probability values yielded by two completely separated CNNs, the first being trained with B-mode ultrasound images and the second being trained using CEUS images. All these methods are detailed below.

(1) **Performing feature level fusion**

In order to perform feature level fusion, the pixel data from the B-mode ultrasound images, respectively from the CEUS images, was directly fused, pursuing to combine the two types of information provided by each image modality, the first one referring to the properties of the tissue with respect to the reflection of the ultrasounds and the second one, to the characteristics of the vessel structure, put into evidence with the aid of the contrast agent. The fusion of the information at this level assumed to perform the arithmetic mean, the weighted mean, and the multiplication between the corresponding pixel values from the two images, respectively. After multiplication of the correspondent pixel values, the result was scaled in the appropriate interval, of [0,255]. The weighted mean was performed with emphasis on the CEUS images, as illustrated in (3):

(3) $$w\_mean\left(i,j\right)=\frac{2\cdot contrast(i,j)+b\_mode(i,j)}{3}$$

**Figure 3:**
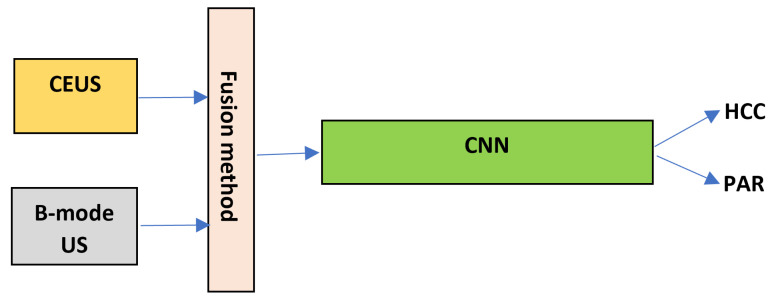
Feature level fusion.

The schema in Figure 3 illustrates this process. In Figure 3, the CNN block stands for the entire CNN network, including the convolutional part, the fully connected layers and the *softmax* layer. *HCC* and *PAR* stand for the output probabilities for the two considered classes, HCC and cirrhotic parenchyma on which HCC had evolved.

(2) **Performing classifier level fusion**

**Figure 4:**
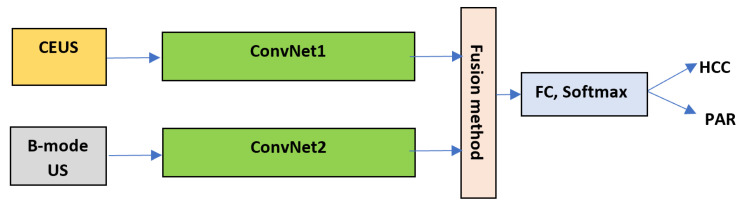
Classifier level fusion.

The classifier level fusion was achieved by separately training the convolutional units of two separate CNN architectures with CEUS, respectively B-mode US data, then providing the fused features to common supervised classifiers, such as fully connected and/or softmax units. This method was adopted as the efficiency regarding the physical resources and execution time was also targeted. In order to perform the fusion of the feature maps provided by each of the CNN structures, the following methods were experimented: the concatenation; the arithmetic mean, the weighted mean and the multiplication, which were performed between the corresponding elements of the linear feature maps (feature vectors), provided by the convolutional units; respectively, the two feature vectors were concatenated and then KPCA was applied for dimensionality reduction. The weighted mean was performed according to Formula (3). This procedure is depicted in Figure 4, where the *ConvNet* blocks stand for the convolutional part of the network (i.e., convolutional, *ReLU*, pooling or batch normalization layers), while the *FC* block stands for the fully connected layers. The two networks involved in this process can have the same architecture or different architectures. When combining different CNN architectures, in the case of multiplication, arithmetic or weighted mean, since the output feature vectors had different sizes, PCA was applied upon the larger feature vector, for dimensionality reduction, in order to obtain the same vector size as that provided by the other network.

(3) **Performing decision level fusion**

**Figure 5:**
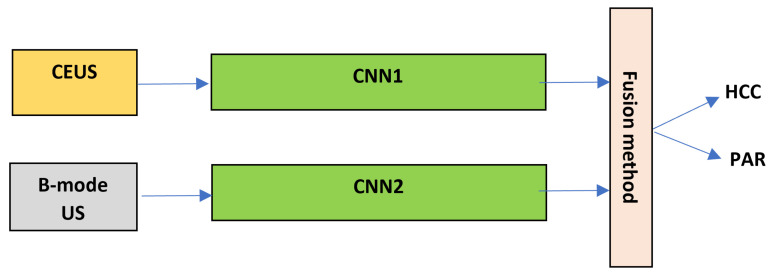
Decision level fusion.

In order to combine the results provided by two completely distinct CNN-based classifiers, the first one being trained using B-mode ultrasound images and the second using CEUS images, a voting procedure was employed, through the computation of the arithmetic mean, respectively weighted mean between the probability values provided by each classifier, as shown in Figure 5. As for the weighted mean, the same procedure, described by the formula (3) was applied. The *CNN* blocks (*CNN1* and *CNN2*) represent the entire network, including the convolutional part, the fully connected layers and the *softmax* layer. *CNN1* and *CNN2* represent either the same CNN architecture or different CNN architectures.

#### 2.2.3. Comparing the CNN Methods with Conventional Approaches

In order to provide a reliable comparison of the CNN performance with that due to the traditional approaches, classical and advanced texture analysis methods were employed for computing potentially relevant textural parameters, able to highlight the visual and physical properties of the tissue. Then, appropriate feature selection methods were applied, in order to determine the set of the relevant textural features, able to best separate between the considered classes. The feature selection techniques were applied as follows: (a.) upon the textural features computed from the B-mode ultrasound images; (b.) upon the textural features derived from the CEUS images; (c.) upon the set of concatenated textural features, resulted from the B-mode ultrasound and CEUS images. The values of the relevant textural features were provided at the entrances of well-known, powerful classifiers. The corresponding methods are detailed within the next paragraphs.

(1) **Texture analysis methods**

Concerning the classical techniques, the autocorrelation index [60] was determined, for assessing the granularity of the tissue. In order to quantify the tissue structure complexity, specific parameters, such as the edge frequency, edge contrast and edge orientation variability [60], the density and frequency of the textural microstructures resulted after the application of the Laws’ convolution filters [61] and the Hurst fractal index [15] were also calculated. Multiresolution features, such as the Shannon entropy, determined after the recursive, twice application of the Wavelet transform, were taken into account also [49]. Concerning the advanced textural features, the Haralick parameters resulted from superior order co-occurrence matrices were included within the feature set. The elements of the generalized, superior order co-occurrence matrix are defined as illustrated within Formula (4):

(4) $$\begin{array}{c} C_{D}\left(f_{1},f_{2},\ldots ,f_{n}\right)=\#\{(\left(x_{1},y_{1}\right),\left(x_{2},y_{2}\right),\ldots ,\left(x_{n},y_{n}\right):\\ f\left(x_{1},y_{1}\right)=f_{1},f\left(x_{2},y_{2}\right)=f_{2},\ldots ,f\left(x_{n},y_{n}\right)=f_{n},\\ |x_{2}-x_{1}\left|=\right|\vec{dx_{1}}\left|,\right|x_{3}-x_{1}\left|=\right|\vec{dx_{2}}\left|,\ldots ,\right|x_{n}-x_{1}\left|=\right|\vec{dx_{n-1}}|,\\ |y_{2}-y_{1}\left|=\right|\vec{dy_{1}}\left|,\right|y_{3}-y_{1}\left|=\right|\vec{dy_{2}}\left|,\ldots ,\right|y_{n}-y_{1}\left|=\right|\vec{dy_{n-1}}|,\\ sgn\left(\left(x_{2}-x_{1}\right)\left(y_{2}-y_{1}\right)\right)=sgn\left(\vec{dx_{1}}\cdot \vec{dy_{1}}\right),\ldots ,\\ sgn\left(\left(x_{n}-x_{1}\right)\left(y_{n}-y_{1}\right)\right)=sgn\left(\vec{dx_{n-1}}\cdot \vec{dy_{n-1}}\right))\} \end{array}$$

According to Formula (4), an element of a superior order, Generalized Co-occurrence Matrix (GCM) computes the number of the n-tuples of pixels which have the values $f_{1}$, $f_{2}$, $f_{3}$,…, $f_{n}$ for a certain feature *f*, being in a spatial distribution defined by the displacement vectors, illustrated in (5):

(5) $$\vec{d}=\left(\left(\vec{dx_{1}},\vec{dy_{1}}\right),\left(\vec{dx_{2}},\vec{dy_{2}}\right),\ldots ,\left(\vec{dx_{n-1}},\vec{dy_{n-1}}\right)\right)$$

In (4), the symbol # stands for the cardinality of the set, referring to the number of elements, each element being represented by an n-tuple of pixels (group of n pixels), having the coordinates $\left(x_{1},y_{1}\right),\left(x_{2},y_{2}\right),\ldots ,\left(x_{n},y_{n}\right)$, always being in a specific spatial configuration, as provided by the displacement vectors, according to (5). A certain local feature, denoted by *f*, is taken into account, being measured in each point, having the values $f_{1},f_{2},\ldots ,f_{n}$, corresponding to each pixel in the n-tuple. This local feature can refer to the intensity value of the pixel, to the value resulted after applying a convolution filter for edge detection, to an edge orientation value, or to a label obtained after applying a clustering algorithm.

In our case, the feature *f* was associated with the intensity values of the pixels, the second and third order GLCM being determined. Thereafter, the corresponding Haralick features were computed, such as the local homogeneity, the energy, the entropy, the correlation, the contrast and the variance, considered able to reveal the differences in heterogeneity, granularity and gray level complexity between the two region of interest types, corresponding to the compared tissue classes [60]. In the case of the second order GLCM, the module of the corresponding displacement vectors was considered as having the value 1, while the direction varied between $0^{\circ }$ and $360^{\circ }$, being always a multiple of $45^{\circ }$. Finally, the Haralick features were computed as the arithmetic mean of the individual values, for each of the GLCM matrices, which corresponded to each combination of parameters. In the case of the third order GLCM, the three considered pixels were either collinear, or they formed a right angle triangle, the current pixel being always in the central position, as described in [9]. As in the previous case, the Haralick features were computed for each configuration, then their values were averaged.

All the textural features were computed on the regions of interest (HCC and PAR patches), independently on orientation, illumination and region of interest size.

(2) **Feature selection methods**

Concerning the feature selection techniques, two representative methods, which provided the best results in our previous work, were taken into account, being shortly presented below:

(a) The **Correlation-based Feature Selection (CFS)** is a feature selection method that evaluates attribute subsets by computing, for each possible subset, a merit, with respect to the class parameter. Thus, the features from the subset were considered relevant if they were strongly correlated with the class parameter and weakly correlated with the other features [62]. CFS was employed together with the Best First Search algorithm, which generated an appropriate set of feature subsets to be assessed [63].
(b) The method of **Gain Ratio Attribute Evaluation** assessed the individual attributes $A_{i}$, i=1,…,m (*m* being the number of attributes), by associating them with a gain ratio. This ratio emphasized the decrease in the class entropy after observing the attribute $A_{i}$, reported to the entropy of $A_{i}$ within the whole dataset [62]. This method was employed in conjunction with the Ranker method [63]. In the case of CFS, the feature subset corresponding to the most increased merit was taken into account, while for the Gain Ratio Attribute Evaluation technique, the first ranked attributes with a gain ratio above 0.15 were considered. The union of the relevant feature subsets, provided by each individual method, was finally considered—the corresponding values being provided as inputs to the conventional classification methods.

(3) **Conventional classification techniques**

Aiming to assess the ability of the textural features for discriminating, in each experimental case, between the two considered classes, HCC and PAR, powerful classifiers and meta-classifiers, which provided the best results in our former experiments, were taken into account, as follows: (a.) the **SVM** classifier, which demonstrated its efficiency in many state-of-the-art approaches [13], with various kernel types: Radial Basis Function (RBF) kernel, respectively polynomial kernel; (b.) the **MLP** classifier, with up to three hidden layers and different number of nodes within each layer, the best such architecture being chosen [49]; (c.) the **AdaBoost** technique in conjunction with the C4.5 algorithm for decision trees, a representative meta-classifier, well known for its performance [49]; (d.) the **Random Forest (RF)** meta-classifier, which employs ensembles of decision trees, in order to gain an increased classification performance [49].

The learning parameters and classifier structures were varied in each case, the best configurations being adopted. In order to assess the *classification performance*, the following metrics, derived from the confusion matrix, were taken into account: the recognition rate (accuracy), the sensitivity, the specificity and the Area under the Receiver Operating Characteristics (AUC). The classification accuracy, also called recognition rate, estimates the number of the correctly classified instances reported to the total number of instances in the training set, performing a global evaluation of the correctness of the classifier result. The True Positive Rate (TPR) or the sensitivity, assesses the numerical fraction between the correctly classified instances from the positive class and the total number of instances from the positive class [61]. Usually, when working with medical data, the positive class is considered that which represents the targeted affection. Thus, the sensitivity estimates the capability of a certain classifier to correctly identify the presence of that affection. The True Negative Rate (TNR) or the specificity assesses the classifier regarding the correct identification of the negative class (associated with the absence of the affection). If the specificity is high, the probability to erroneously diagnose a certain disease is low. Both the sensitivity and the specificity are important in the context of cancer diagnosis, leading to an increased probability to reach the disease when it is present, respectively to a low probability to send the patient to a harmful treatment when it is not actually needed. The ROC curve represents the plot of the *sensitivity* versus *1-specificity*, which is equivalent with plotting the TPR, represented on the vertical axis, versus the FPR represented on the horizontal axis. Thus, ROC illustrates the trade-off between the true positives, standing for the benefits and the false positives, representing the costs, the performance of a certain classifier being in direct relation with the area under this plot [61].

## 3. Experiments

The CNN techniques were employed within the Matlab 2020 environment, using the Deep Learning Toolbox [64]. During training, the Stochastic Gradient Descent with Momentum (SGDM) strategy was adopted, with a small enough learning rate (0.0002), a momentum of 0.1, a minibatch size of 25, the duration of the training process being set to 70 epochs. The values of the above-mentioned hyperparameters were chosen to achieve a refined and efficient learning process and simultaneously to avoid overtraining (the learning rate, momentum and the number of epochs), also taking into account the memory requirements (the minibatch size). The corresponding functions of the Deep Learning Toolbox and the specific CNN structures, *squeezenet*, *googlenet*, *vgg16*, *resnet18* and *densenet201* were exploited. These networks were pretrained using the ImageNet dataset; then, the training was refined using the specific data from the B-mode ultrasound and CEUS images. In addition, the last layer of these networks was reshaped to provide only two output features, corresponding to the HCC and PAR classes. The method of KPCA was employed with the aid of the Matlab–Kernel–PCA toolbox [65]. The linear, third degree polynomial and Gaussian kernels were experimented.

ROC analysis was performed in Matlab also. In order to perform ROC curve representation, the *perfcurve* function was employed. This function needed three arguments: the label vector, the score vector and the reference to the positive class. The labels were converted to numeric values of type double, the score vector contained the probabilities for the positive class, a single numeric value being employed in order to refer to the positive class. The score vector resulted as output of the *classify* or *predict* functions. In order to simultaneously determine multiple ROC curves in a time-efficient manner, the trained versions of the CNN classifiers, previously saved on the disk, were loaded, the test set was provided at the input of each classifier in order to determine the score vector, then the *perfcurve* function was applied appropriately. For decision level fusion, the arithmetic mean of the two score vectors, corresponding to each of the combined CNN classifier, was computed, then this value was provided as the second argument of the *perfcurve* function. The ROC curves corresponding to multiple classifiers were superimposed on the same figure, with the aid of the *plot* functions and *hold on* statements.

The textural attributes (41 features) determined by our Visual C++ software modules were included in the experiments.

The feature selection techniques and the conventional classification methods were employed using the Weka 3.8. library [63]. Regarding the feature selection methods, the CfsSubsetEval(CFS) method was implemented with BestFirst search, while the GainRatioAttributeEval was employed in conjunction with the Ranker search technique. As for the conventional classifiers, the John Platt’s Sequential Minimal Optimization (SMO) algorithm [63] for SVM was assessed—the best performance resulting for the polynomial kernel, of 3rd or 5th degree. The MLP technique was implemented as well, a momentum of 0.8, a learning rate of 0.2 and a training time of 500 epochs being considered, in order to achieve a refined learning process, to avoid overtraining, and to rich convergence. Different architectures, with one, two and three hidden layers, each of them with a number of nodes provided by the arithmetic mean between the number of classes and the number of features were assessed. The AdaBoost metaclassifier was evaluated for 100 iterations, in conjunction with the J48 method, the equivalent of the C4.5 algorithm in Weka. The Random Forest (RF) method of Weka, with 100 iterations and batch size 100, which provided the best results in our former experiments [49], was experimented as well.

All these experiments were performed on a computer having an i7 processor of 2.60 GHz, 8 GB of internal (RAM) memory and an Nvidia Geforce GTX 1650 Ti GPU. Regarding *the performance evaluation strategy*, for the CNN methods, 60% of the data constituted the training set, 15% of the data stood for the validation set and 25% of the data was included in the test set. Concerning the traditional classification techniques, 75% of the data was included in the training set and 25% of the data constituted the test set.

## 4. Results

### 4.1. CNN Assessment on CEUS and B-Mode Ultrasound Images

Firstly, the proposed CNN architectures were assessed on CEUS and B-mode ultrasound images, separately. The results that correspond to the CEUS images are depicted in Table 1. The highest value for each column is emphasized with bold characters. As can be noticed, the best accuracy, of 91.6%, the best sensitivity, of 93.5%, the highest AUC, of 92.04%, were obtained for the ResNet architecture, the best specificity, of 94.1%, resulting for the DenseNet architecture. The GoogLeNetV1 classifier achieved an accuracy of 86.7%, which was superior to the accuracy of the SqueezeNet and standard GoogLeNet architectures, but also closed to that of VGGNet, while the training time of 136 min and 48 s was more decreased than the training time of VGGNet (200 min).

The results obtained when providing the B-mode ultrasound images at the CNN inputs are depicted in Table 2. The best accuracy, of 90.5%, was obtained in the case of the ResNet architecture, the best sensitivity, of 95.2%, and the best AUC, of 90.4%, resulted in the case of VGGNet, while the best specificity, of 94.3%, resulted in the case of the GoogLeNetV1 architecture. It can be also remarked that, in the case when providing the CEUS images at the CNN inputs, the results were always superior than in the situation when the B-mode ultrasound images were employed.

### 4.2. CNN Assessment on Combined CEUS and B-Mode Ultrasound Images

#### 4.2.1. Feature Level Fusion

The results achieved in the case of feature level fusion are illustrated within Table 3. It results that in the case when the arithmetic mean was employed, the best accuracy, of 93.2%, and the best AUC, of 93.2%, were obtained for DenseNet, the best sensitivity, of 93.6%, was obtained for GoogLeNet, the best specificity, of 95%, was achieved for VGGNet. When considering the weighted mean as the fusion method, the best accuracy, of 92.6%, and the best AUC, of 93.01%, were obtained for DenseNet, the highest senitivity, of 96.1%, resulted for ResNet, while the highest specificity, of 96.3%, resulted for GoogLeNetV1. In most of the cases, for the SqueezeNet, GoogLeNet and VGGNet classifiers, the classification accuracy values resulted for the weighted mean strategy were superior to those obtained for the arithmetic mean strategy, for the same classifiers, but in the case of the CNN architectures involving residual connections, the accuracy values were superior when employing the arithmetic mean combination method. However, no significant differences were noticed between the results achieved in the case of arithmetic mean employment and weighted mean employment, respectively. When considering the multiplication as a feature level fusion method, the highest accuracy, of 94.7%, the best sensitivity, of 96.4%, as well as the most increased AUC, of 94.89%, were obtained for the DenseNet technique, while the most increased specificity, of 96.1%, resulted for the ResNet architecture. As can be noticed, the results obtained for multiplication are superior to the other feature level fusion cases. Thus, the multiplication emphasized those pixels which had, in both B-mode and CEUS images, either increased or decreased intensity values, corresponding to actively growing tumour tissue or to the necrosis regions. Eloquent examples of HCC and PAR patches, obtained in each case of feature level fusion, by performing the above-mentioned operations upon the B-mode ultrasound and CEUS images, are illustrated in Appendix A.

#### 4.2.2. Classifier Level Fusion

In the case of classifier level fusion, for each considered CNN architecture, the features obtained at the output of the last layer, preceding the classification layers (*fully connected* or *softmax*), were usually taken into account, as described below.

(a) In the case of *SqueezeNet*, the following feature vectors were taken into account: the output of the last layer, “pool10” having the size of 2; the feature vector obtained at the output of the previous layer, “relu_conv10”, of size 392 (14×14×2); the concatenation of these two feature vectors (size 394).
(b) In the case of *GoogLeNet*, the feature vector of size 1024 (1×1×1024) obtained at the output of the “pool5-drop_7X7_s1” layer, preceding the fully connected layer, was considered; the same for the modified version of GooLeNet, denoted by *GoogLeNetV1*, but the output was of size 528.
(c) In the case of *ResNet18*, the output of the “pool5” layer, of size 512 (1×1×512), preceding the fully connected layer, was taken into account.
(d) In the case of *VGG16*, the output of the “drop7” dropout layer, of size 4096 (1×1×4096), preceding the fully connected layer, was retained.
(e) As for *DenseNet201*, the output of the “avg_pool” layer, of size 1920 (1×1×1920), preceding the fully connected layer, was considered.

Thereafter, the supervised classification layer was implemented as a *feedforward network*, *softmax* function, or *fitececoc* function, the Matlab equivalent of SVM, the best obtained results being depicted.

The values of the corresponding performance parameters are presented within Table 4. The highest values obtained for each performance parameter, for each fusion method, are emphasized. Thus, in most of the situations, the best performance resulted for the concatenation, respectively for the arithmetic and weighted mean.

KPCA led to the best values of the performance parameters when either the *linear kernel* or the *3rd degree polynomial kernel* was considered. In the case of SqueezeNet, when taking into account the feature vector obtained as the output of the “relu_conv10” layer, the highest specificity, of 93.61%, resulted in the case of KPCA, for the 3rd degree polynomial kernel. In addition, a satisfying classification performance (accuracy above 83%), in the case when employing KPCA as a combination method, resulted for the SqueezeNet, GoogLeNet and VGGNet architectures.

A more detailed comparison regarding the combination methods employed in order to perform classifier level fusion is depicted in Appendix B.

Regarding the comparison between the considered CNN architectures, the best accuracy, of 97.25%, the best specificity, of 97.58%, and the best AUC, of 97.22%, resulted for the hybrid multimodal-combined classifier, obtained by combining a DenseNet branch, pretrained with CEUS images, respectively a ResNet branch, pretrained with B-mode ultrasound images, when the *concatenation* fusion method was implemented, while the best sensitivity, of 97.11%, was achieved for the alternative hybrid combined multimodal classifier, resulted from a ResNet branch, pretrained with CEUS images, respectively from a DenseNet branch, pretrained with B-mode ultrasound images, when the *concatenation*, was adopted. Thus, the fusion, at classifier level, between the best performing architectures, DenseNet and ResNet, was experimented, leading to very good results, as illustrated in Table 4. In the case of the new GoogLeNetV1 architecture, an obvious increase in performance was noticed for classifier level fusion, in the cases of arithmetic mean, concatenation, and multiplication. The values of the classification performances in these cases was superior to those obtained for GoogLeNet and SqueezeNet, while in the cases of arithmetic mean, weighted mean and multiplication, the values of these parameters were superior to the values resulted for ResNet, in the same situations. These results were superior to those attained separately on CEUS and B-mode ultrasound images, when applying the considered CNN-based techniques, but also to the feature level fusion case.

#### 4.2.3. Decision Level Fusion

The values of the classification performance parameters corresponding to decision level fusion are depicted within Table 5. It can be observed that the performance was the best, in most of the situations, when the arithmetic mean was adopted as a decision level combination technique. The best performing methods, ResNet and DenseNet, were also combined with each other, constituting the branches of the fused classifiers, in the following manner: the DenseNet classifier trained on CEUS images was combined with the ResNet classifier trained on B-mode ultrasound images and also the ResNet classifier trained on CEUS images was combined with the DenseNet classifier trained on B-mode ultrasound images. Thus, when taking into account the arithmetic mean as a decision level fusion method, in the case when the same architecture was involved in the decision level fusion, the best accuracy, of 97.49%, the best sensitivity, of 96.59%, the highest specificity, of 98.24%, as well as the highest AUC, of 97.43%, were achieved for DenseNet. However, in the situation when fusing the DenseNet classifier trained on CEUS images with the ResNet classifier trained on B-mode ultrasound images, an accuracy of 98.20%, a sensitivity of 98.16%, a specificity of 98.24% and an AUC of 98.20% were obtained, which were superior to the values of the performance parameters obtained in all the other cases. When the weighted mean was considered as the decision level fusion technique, the most increased accuracy, of 96.77%, the highest sensitivity, of 97.64%, as well as the highest AUC, of 96.85%, resulted for the case when the DenseNet CNN architecture was combined with itself; the best specificity, of 99.54%, was attained when fusing the GoogLeNetV1 classifier with itself. The overall performance results in this case were superior to those achieved when providing the CEUS, respectively the B-mode ultrasound images separately, at the entrances of the CNN-based classifiers. In addition, the best values of the classification performance parameters were higher than those resulted in the case of feature level fusion, as well as in that of classifier level fusion.

### 4.3. Comparisons with Conventional Approaches

#### 4.3.1. Texture Analysis and Recognition on CEUS, and B-Mode Ultrasound Images

The values of the classification performance parameters, resulted after applying texture analysis methods in conjunction with conventional classifiers, upon CEUS images, are presented in Table 6. As can be seen in Table 6, the best accuracy, of 82.1%, the best sensitivity, of 91.5%, as well as the highest specificity, of 72.5%, resulted for the AdaBoost metaclassifier combined with decision trees (J48), while the best AUC, of 88.1%, was obtained for the RF classifier. These results are inferior to those achieved with the CNN-based techniques on the same CEUS dataset.

The classification performance achieved when providing, at the entrances of the conventional classifiers, the values of the relevant textural features derived from the B-mode ultrasound images are illustrated in Table 7. Thus, the best accuracy, of 75.1%, the highest sensitivity, of 90.1%, and the most increased AUC, of 83%, were attained in the case of the RF classifier, while the best specificity, of 60.3%, resulted for the MLP classifier. As can be noticed, these results are inferior to those obtained on the same B-mode ultrasound images, when employing the CNN-based techniques, being also inferior to those achieved in the case of the CEUS images, when employing the conventional approach.

#### 4.3.2. Texture Analysis and Recognition on Combined CEUS and B-Mode Ultrasound Images

The values corresponding to the classification performance parameters, obtained after applying the conventional approach described within Section 2.2.3, upon the combination of CEUS and B-mode ultrasound images, are depicted in Table 8. The highest accuracy, of 87.1%, as well as the best sensitivity, of 98.2%, were achieved in the case of the AdaBoost metaclassifier, the best specificity, of 76.2%, resulted for the MLP classifier, while the most increased AUC value, of 95.1%, was obtained for the RF classifier. These results were generally inferior to those obtained for the same dataset, when applying CNN-based techniques, but they were superior to those obtained when employing the considered conventional approach, on CEUS, respectively on B-mode ultrasound images, separately. However, the best accuracy, of 87.1%, obtained for the AdaBoost metaclassifier, was comparable to that resulted in the case of SqueezeNet, when the combination methods of arithmetic mean and weighted mean, respectively, were applied for feature level fusion. Additionally, the best sensitivity, of 98.2%, resulted for the same conventional classifier, overpassed the sensitivity values obtained by employing the CNN technique, while the AUC value was comparable with those achieved for the CNN-based method, in all the experimental situations. Regarding the relevant textural features resulted in this case, those computed based on the CEUS images played a major role, such as: the Haralick parameters derived from the GLCM matrices, as well as the entropy computed after the recursive application of the Wavelet transform, emphasizing the complex, chaotic character of the HCC tissue, also differences in structural complexity between the HCC and PAR classes. The autocorrelation index was also part of this relevant textural features set, indicating differences in granularity between the two considered tissue types.

## 5. Discussion

**Figure 6:**
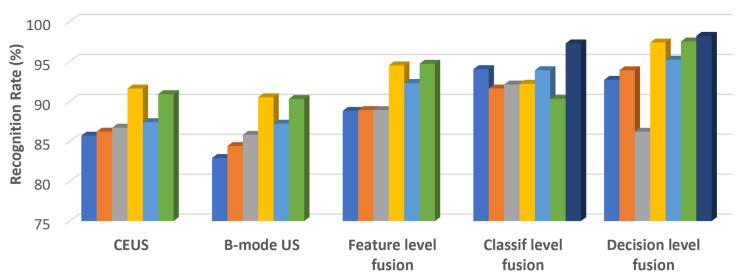
Comparison of the classification accuracy values achieved by the considered CNN architectures, for each fusion strategy.

As can be seen in the results from the previous section, the combination between the CEUS and B-mode ultrasound images led to significant classification improvement, in comparison to the case when the CEUS and the B-mode ultrasound images were separately taken into account, respectively. The best classification performance resulted in the case of the hybrid multimodal combined classifiers, when performing decision level fusion, by combining the DenseNet branch, pretrained using CEUS images, with the ResNet branch, pretrained with B-mode ultrasound images. A classification accuracy above 98% was achieved in this case. A detailed comparison regarding the best values of the classification accuracy, obtained for each fusion level, for each employed CNN architecture or multimodal combined classifier, is depicted in Figure 6. Within this figure, it can be noticed that the accuracy values obtained for the CEUS images were slightly better than those resulted for the B-mode ultrasound images, while the adopted combination schemes were obviously superior to these cases from the accuracy point of view. Thus, the best performance resulted for the decision level fusion schemes, followed by the classifier level fusion schemes, then by the feature level fusion schemes. Regarding the performance of the CNN architectures, the structures involving residual connections, ResNet, and DenseNet, respectively, led to the best results. Additionally, the hybrid multimodal combined classifiers, resulted through the combination between the DenseNet and ResNet branches generated a significant performance improvement, both for classifier and decision level fusion, especially when the DenseNet branch was pretrained with CEUS images and the ResNet branch was pretrained with B-mode ultrasound images. The GoogLeNetV1 architecture provided satisfying accuracy, slightly better than the usual GoogLeNet architecture and sometimes close to those provided by the VGGNet architecture, the specificity having very high values in all these cases. An obvious increase in performance for this architecture resulted in the case of classifier level fusion, when the outputs of different inception modules, for both CEUS and B-mode ultrasound images, were fused in various manners.

Within Table 9, the values of the classification performance parameters assessed on the validation set, for our best performing CNN classifiers, are depicted. These values are almost similar to those illustrated previously (within Section 4), which were evaluated on the test set, confirming the already resulted performance, for feature level fusion, classifier level fusion and decision level fusion, respectively.

**Figure 7:**
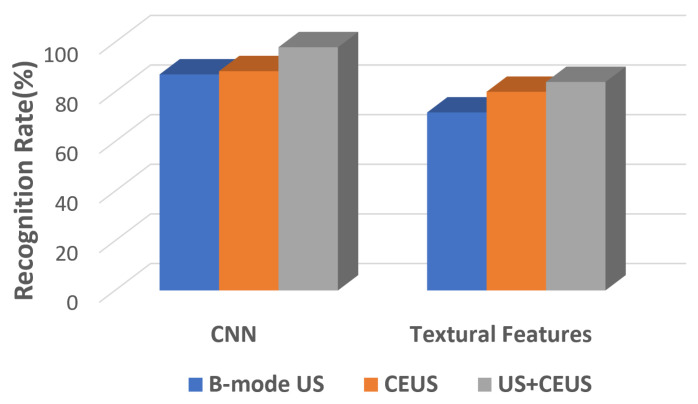
Comparison of the classification accuracy values achieved in each case by the CNN-based techniques or through the conventional approach.

The overall comparison between the best accuracy values obtained for the CNN-based classifiers, respectively those resulted for the classical approach, involving advanced texture analysis methods and conventional classifiers, is depicted in Figure 7. It can be observed that, in the case of the CNN classifier, the best obtained accuracy was superior, in all cases, for B-mode ultrasound images and CEUS images separately, respectively for the fusion between the CEUS and B-mode ultrasound images. For each class of techniques, the best accuracy resulted in the case of the fusion between CEUS and B-mode ultrasound images, followed by the case when only the CEUS images were employed, then by the situation when the B-mode ultrasound images, or the relevant textural features derived from these images were provided at the classifier inputs.

**Figure 8:**
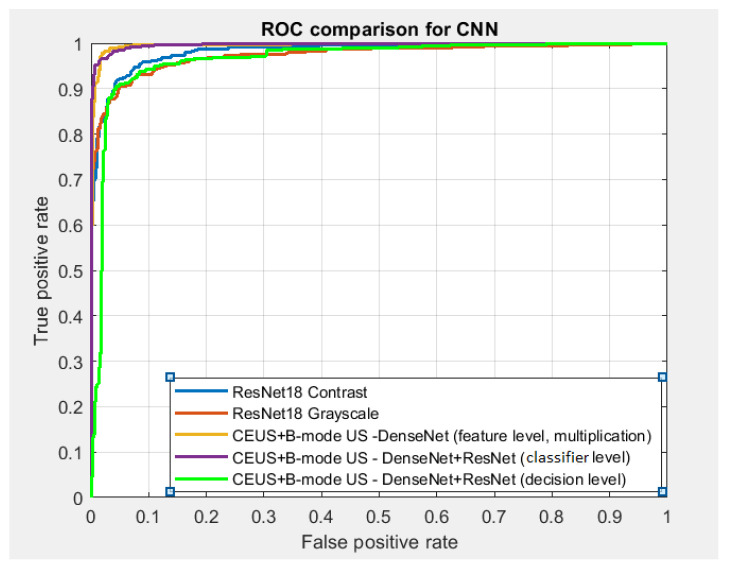
Comparison of the ROC curves for the best classifiers.

Within Figure 8, the ROC curves for the best performing classifier, for each considered experimental case (CEUS and B-mode ultrasound image with separate employment; feature level fusion, classifier level fusion and decision level fusion), can be compared. Thus, the best ROC curves, having the most increased area below, were those corresponding to feature level fusion by multiplication (DenseNet) and to classifier level fusion (DenseNet + ResNet), respectively. Additionally, the ROC curve corresponding to the situation when only the CEUS images were employed, with the aid of the ResNet architecture, was superior to that corresponding to the case when the B-mode ultrasound images were separately exploited (ResNet), while the ROC curve corresponding to the decision level fusion case (DenseNet + ResNet) was better than the first.

**Figure 9:**
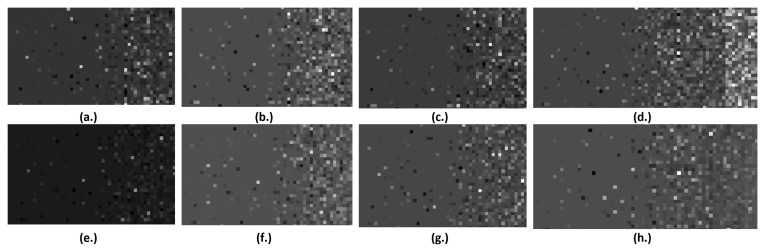
The activation maps of the best performing classifiers: (**a.**–**d.**)—the class of HCC for B-mode US and CEUS input (ResNet), respectively for feature level fusion (DenseNet, multiplication) and classifier level fusion (DenseNet + ResNet); (**e.**–**h.**)—the class of PAR, for the same cases.

The activation maps corresponding to the best performing classifiers, generated for both HCC and PAR patches, are illustrated in Figure 9. They were achieved by taking into account the output of the last layer (dropout, pooling, ReLu or combined), preceding the fully connected or *softmax* layer, which was transposed into a grayscale image of appropriate size. Thus, in the cases when the B-mode ultrasound and CEUS images were separately employed, the output, a feature vector of length 512, was provided by the last pooling layer of the ResNet architecture (“pool5”) and was transposed in a grayscale image having 16×32 pixels in size; in the case of feature level fusion, as the best performing classifier was DenseNet, the output was acquired from the last average pooling layer (“avg_pool”), a vector with 1920 elements, which was transposed into a grayscale image having 32×60 pixels in size; in the case of classifier level fusion, the displayed image, having 32×76 pixels in size, resulted from the concatenation of the output of DensNet (“avg_pool”), pretrained with CEUS images, with the output of ResNet (“pool5”), pretrained with B-mode ultrasound images. It can be remarked that, in the case of HCC, the corresponding intensity pattern is more heterogeneous and has a better contrast, in comparison with the case of the PAR class, these differences being more emphasized in the case of B-mode ultrasound images, respectively in that of classifier level fusion. They confirm the a priori known properties, of in-homogeneity and structural complexity, of the HCC tumor tissue. These maps can stand as the basis for a newly defined model of HCC, following the previously defined textural model, described in [9].

### Comparisons with Other State-of-the-Art Results

Considering the relevant state-of-the-art approaches that concern the automatic recognition within medical images using multiple image modalities, illustrated within Section 1.2, our solution mostly resembles the one presented in [44]. Thus, in [44], the authors perform only classifier level fusion, in order to automatically recognize breast cancer, based on five types of histological and imunohistochemical image data. Three standard CNN architectures were taken into account: VGG16, InceptionV3, and ResNet50, respectively. As stated in [44], the best performing solution consisted of training the CNNs on the five image modalities, which were then combined, at classifier level, by applying the PCA method on the concatenated vector of the resulted activations. The result of PCA was then provided as input to a LDA classifier. Concerning the combined classifiers presented in [44], each combination always involved the same CNN architecture. In order to compare our solution with the previously mentioned approach, in the same conditions, we employed the InceptionV3, ResNet50 and VGG16 architectures from the Matlab Deep Learning toolbox [64], which were pretrained on our B-mode ultrasound and CEUS data. The corresponding activation maps were then concatenated and the linear PCA method was applied, the result being provided as argument to the *fitcdiscr* function, the Matlab equivalent of LDA classifier. The performance was assessed on the test set and compared with the best performance resulted in our work, while performing feature level fusion, classifier level fusion and decision level fusion. This comparison was depicted within Table 10. Our current approach was also compared with our previous approach, presented in [48], where we evaluated, on a subset of the current dataset, the performance resulted by applying the SAE classifier on combined CEUS and B-mode ultrasound images. The best performance was achieved in the case of feature level fusion, when providing, at the input of SAE, the relevant textural features, derived from the concatenated textural attributes, corresponding to both B-mode ultrasound and CEUS images. The comparison between the performance of our approach and that of the above mentioned approach, assessed on the current dataset, is depicted in Table 10. We can notice that in the case of the multimodal classifiers corresponding to the approach presented in [44], the values of the classification performance parameters were comparable to those obtained for the classifier level fusion, when applying KPCA with a 1st or 3rd degree polynomial kernel, as illustrated in Table 4. However, as can be seen in Table 4, concerning the classifier level fusion, a better performance was achieved when employing concatenation, arithmetic or weighted mean as combination methods, the best result of this category being also illustrated within Table 10. The values of the classification performance parameters obtained in the case of decision level fusion, when combining the DenseNet classifier, trained with CEUS images, with the ResNet classifier, trained with B-mode ultrasound images, were superior, as well, to those achieved for the methods described in [44], employed on our dataset. In addition, in the case of feature level fusion, when employing multiplication, the classification performance was superior to that resulted for the methods presented in [44]. Concerning the approach presented in [48], the best resulted performance was inferior to that resulted in the context of the current research.

Our approach can be also compared with the approach described in [43], regarding both the methodology and applicability domain. However, this approach evaluated dynamic sequences of B-mode ultrasound and CEUS images. The employment of dynamic image sequences and of the corresponding temporal information is part of our future research objectives. We can conclude that the classification performance achieved through our approach is comparable, even superior, to the state-of-the-art approaches previously presented, although not all the evaluations have been performed of the same dataset.

## 6. Conclusions

The proposed methodology, as well as the analysis of the experimental results, demonstrated that the CEUS images and especially the combination between B-mode ultrasound and CEUS images led to a considerable classification performance improvement, in comparison to the case when the B-mode ultrasound images were separately employed. The newly defined multimodal combined classifiers finally led to a classification accuracy above 97%, generally better than the performance reported in the state of the art of the domain. As for the future work, the dataset will be enhanced, by gathering more data and by also adding the class of benign liver tumors (e.g., hemangioma). More complex CNN architectures, such as those of Inception and InceptionResNet type, as well as other appropriate combination methods, such as DCCA [34], are also planned to be experimented. The problem of insensitivity to changes in the image acquisition settings that induce variations in image resolution will also be approached. Another future research intention is to take into account also elastographic images, in order to combine them with the existing dataset.

## Figures and Tables

**Table 1:** The values of the performance parameters obtained when providing the CEUS images at the Convolutional Neural Network (CNN) inputs.

CNN	Acc (%)	Sens (%)	Spec (%)	AUC (%)
**SqueezeNet**	85.7	80.5	91.4	86.38
**GoogLeNet**	86.2	86.4	86.1	86.25
**GoogLeNetV1**	86.7	80.9	91.5	86.23
**ResNet**	**91.6**	**93.5**	90.5	**92.04**
**VGGNet**	87.4	85.8	88.9	87.39
**DenseNet**	90.9	86.9	**94.1**	90.71

**Table 2:** The values of the performance parameters obtained when providing the B-mode ultrasound images at the CNN inputs.

CNN	Acc (%)	Sens (%)	Spec (%)	AUC (%)
**SqueezeNet**	82.9	91.2	80.7	86.35
**GoogLeNet**	84.4	89.3	82.9	86.25
**GoogLeNetV1**	85.8	67.7	**94.3**	83.86
**ResNet**	**90.5**	84.3	94	89.52
**VGGNet**	87.2	**95.2**	84.7	**90.4**
**DenseNet**	90.3	84.3	93.5	89.23

**Table 3:** The values of the classification performance parameters for CNNs when performing feature level fusion.

Fusion	CNN	Acc (%)	Sens (%)	Spec (%)	AUC (%)
**Arithmetic** **mean**	**SqueezeNet**	87	88.7	85.2	87
	**GoogLeNet**	87.6	**93.6**	80.9	87.86
	**GoogLeNetV1**	87.9	84.5	85.1	84.80
	**ResNet**	92.6	91.2	94.2	92.74
	**VGGNet**	91.6	89	**95**	92.15
	**DenseNet**	**93.2**	93.4	93	**93.2**
**Weighted** **mean**	**SqueezeNet**	87.5	88.1	87.1	87.6
	**GoogLeNet**	88.9	88.1	89.7	88.9
	**GoogLeNetV1**	88.4	67.1	**96.3**	84.65
	**ResNet**	90.9	**96.1**	86.6	91.73
	**VGGNet**	92.3	89.1	94.2	91.76
	**DenseNet**	**92.6**	95.9	89.8	**93.01**
**Multiplication**	**SqueezeNet**	88.8	89.5	88.2	88.6
	**GoogLeNet**	88.9	91.2	87.1	89.22
	**GoogLeNetV1**	88.9	70.4	95.3	85.02
	**ResNet**	94.5	92.5	**96.1**	94.36
	**VGGNet**	92.3	88.7	95.4	92.24
	**DenseNet**	**94.7**	**96.4**	93.3	**94.89**

**Table 4:** The values of the classification performance parameters for CNNs when performing classifier level fusion.

CNN	Fusion	Acc (%)	Sens (%)	Spec (%)	AUC (%)
**SqueezeNet**	**Concatenation**	93.77	91.34	95.81	93.66
“pool10”	**Arithm.mean**	92.57	90.55	94.27	92.47
	**Weight.mean**	**94.13**	**91.86**	**96.04**	**94.03**
	**Multiplication**	88.38	80.58	94.93	88.55
**SqueezeNet**	**Concatenation**	88.02	87.93	88.1	88.02
“relu_conv10”	**Arithm.mean**	88.62	86.09	90.75	88.50
	**Weight.mean**	**89.82**	**91.86**	88.11	**90.04**
	**Multiplication**	83.71	82.41	84.80	83.63
	**KPCA**(Poly 3rd dgr)	87.78	80.84	**93.61**	87.84
**SqueezeNet**	**Concatenation**	87.90	88.45	87.44	87.95
“relu_conv10”	**Arithm.mean**	**90.06**	**89.50**	90.53	**90.02**
+ “pool10”	**Weight.mean**	89.46	87.66	**90.97**	89.36
	**Multiplication**	84.79	83.99	85.46	84.73
	**KPCA**(Poly 3rd dgr)	89.46	87.93	90.75	89.37
**GoogLeNet**	**Concatenation**	90.1	86.3	**96**	**91.2**
“pool5_drop_7x7_s1”	**Arithm.mean**	89.1	88.2	90.3	89.2
	**Weight.mean**	**91.6**	**95.6**	86.9	82.3
	**Multiplication**	84.6	91	76.9	84.4
	**KPCA** (Linear)	83.7	81.1	85.9	83.2
**GoogLeNetV1**	**Concatenation**	91.86	91.08	92.51	91.8
“pool5_drop_7x7_s1”	**Arithm.mean**	**92.1**	**91.34**	92.73	**93.75**
	**Weight.mean**	90.06	90.03	90.09	90.06
	**Multiplication**	91.74	90.29	**92.95**	91.89
	**KPCA** (Linear)	75.3	80.3	71.0	65.79
**ResNet**	**Concatenation**	**92.2**	**90.9**	**93.9**	**92.1**
“pool5”	**Arithm.mean**	87.3	89.9	84.5	87.3
	**Weight.mean**	81.9	83.3	80.3	82.3
	**Multiplication**	88.9	88.8	88.9	89.2
	**KPCA** (Linear)	76.9	80.1	74.2	77.1
**VGGNet**	**Concatenation**	92.8	**96.9**	87.9	92.75
“drop7”	**Arithm.mean**	**93.9**	96	91.3	**93.75**
	**Weight.mean**	92	94.1	89.5	91.89
	**Multiplication**	92.46	92.39	**92.51**	92.45
	**KPCA** (Linear)	90.7	90.8	90.5	90.65
**DenseNet**	**Concatenation**	87.7	87.9	**87.4**	87.80
“avg_pool”	**Arithm.mean**	**88.6**	**91**	85.8	**88.50**
	**Weight.mean**	80.23	60.12	83.45	73.04
	**Multiplication**	81.4	80.19	82.5	81.36
	**KPCA** (Linear)	75.9	72.3	72.8	72.55
**DenseNet + ResNet**	**Concatenation**	**97.25**	**96.85**	**97.58**	**97.22**
“avg_pool”	**Arithm.mean**	85.12	84.25	80.65	95.75
+”pool5”	**Weight.mean**	80.23	60.12	83.45	73.04
	**Multiplication**	81.4	80.19	82.5	81.36
	**KPCA** (Linear)	75.9	72.3	72.8	72.55
**ResNet + DenseNet**	**Concatenation**	**96.53**	**97.11**	**96.04**	**96.58**
“pool5”	**Arithm.mean**	80.26	84.78	63.91	93.75
+”avg_pool”	**Weight.mean**	80.11	78.47	81.12	79.82
	**Multiplication**	80.6	78.8	81.53	80.19
	**KPCA** (Linear)	73.1	74.2	73.1	73.65

**Table 5:** The values of the classification performance parameters for CNNs when performing decision level fusion.

Fusion	CNN	Acc (%)	Sens (%)	Spec (%)	AUC (%)
**Arithmetic** **mean**	**SqueezeNet**	92.69	91.08	94.05	92.6
	**GoogLeNet**	93.89	91.08	96.26	93.78
	**GoogLeNetV1**	86.19	65.35	98	85.45
	**ResNet**	97.37	96.33	98.24	97.3
	**VggNet**	95.21	92.13	97.8	95.11
	**DenseNet**	97.49	96.59	**98.24**	97.43
	**DenseNet + ResNet**	**98.20**	**98.16**	**98.24**	**98.20**
	**ResNet + DenseNet**	96.77	95.01	**98.24**	96.67
**Weighted** **mean**	**SqueezeNet**	91.62	91.08	92.07	91.58
	**GoogLeNet**	92.10	89.76	94.05	91.99
	**GoogLeNetV1**	85.45	56.73	**99.54**	82.10
	**ResNet**	95.81	95.01	96.48	95.75
	**VggNet**	92.93	91.08	94.49	92.83
	**DenseNet**	**96.77**	**97.64**	96.04	**96.85**
	**DenseNet + ResNet**	92.81	95.8	90.31	93.18
	**ResNet + DenseNet**	95.45	93.44	97.14	95.35

**Table 6:** The values of the classification performance parameters for texture analysis methods combined with conventional classifiers, obtained on CEUS images.

Classifier	Acc (%)	Sens (%)	Spec (%)	AUC (%)
**RF**	79.25	90.7	66.7	**88.1**
**SVM(poly 3rd dgr)**	79.3	89.1	69.8	79.3
**MLP**	79.5	88.7	70.3	86.4
**AdaBoost + J48**	**82.1**	**91.5**	**72.5**	87

**Table 7:** The values of the classification performance parameters for texture analysis methods combined with conventional classifiers, obtained on B-mode ultrasound images.

Classifier	Acc (%)	Sens (%)	Spec (%)	AUC (%)
**RF**	**75.1**	**90.1**	60.2	**83**
**SVM(poly 5th dgr)**	65.3	88.2	50.2	65.9
**MLP**	73.1	85.4	**60.3**	75.2
**AdaBoost + J48**	73.4	87.2	59.7	77.8

**Table 8:** The values of the classification performance parameters for texture analysis methods combined with conventional classifiers, obtained on combined CEUS and B-mode ultrasound images.

Classifier	Acc (%)	Sens (%)	Spec (%)	AUC (%)
**RF**	84.35	95.6	73.1	**95.1**
**SVM(poly 1st dgr)**	83.11	94.0	72.1	83.2
**MLP(a)**	81.5	87	**76.2**	90.2
**AdaBoost + J48**	**87.1**	**98.2**	74.1	93.3

**Table 9:** The values of the performance parameters obtained on the validation set for the best performing multimodal classifiers.

Img. Modality	CNN Classifier	Acc (%)	Sens (%)	Spec (%)	AUC (%)
**CEUS**	**ResNet**	92.02	89.52	94.12	91.91
**B-mode US**	**ResNet**	90.95	88.07	92.43	90.33
**Feature level** **fusion**	**DenseNet** (multiplication)	93.7	97.2	90.8	94.18
**Classifier level** **fusion**	**DenseNet + ResNet** (concatenation)	97.21	95.20	98.90	97.11
**Decision level** **fusion**	**DenseNet + ResNet** (arithmetic mean)	98	96.94	98.90	97.94

**Table 10:** Comparison with state-of-the-art results.

Multimodal Classifier	Acc (%)	Sens (%)	Spec (%)	AUC (%)
**InceptionV3 + PCA-LDA [44]**	73.4	66.1	79.5	73.22
**ResNet50 + PCA-LDA [44]**	78.7	82.2	75.8	79.12
**VGG16 + PCA-LDA [44]**	91.7	91.9	91.6	91.75
**Textural Features + SAE [48]**	90.08	85.1	94.2	89.9
**DenseNet201—feature level** **multiplication** (crt. work)	94.7	96.4	93.3	94.89
**DenseNet201 + ResNet18—classif. level** **concatenation** (crt. work)	97.25	96.85	97.58	97.22
**DenseNet201 + ResNet18—decision level** **arithm. mean** (crt. work)	**98.25**	**98.16**	**98.24**	**98.2**

**Figure A1:**
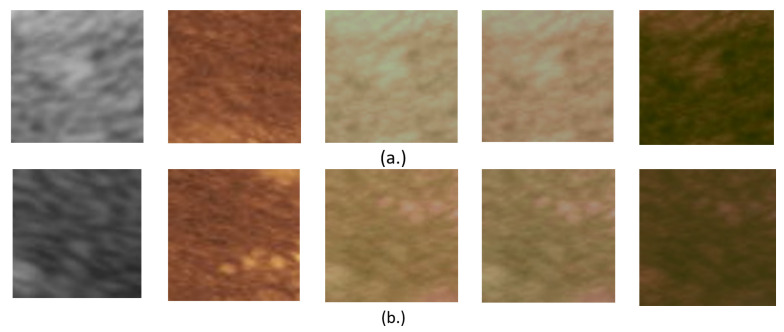
Examples of (**a.**) HCC and (**b.**) PAR patches, in the following cases (left -> right): the B-mode ultrasound image; the CEUS image; the arithmetic mean of the B-mode ultrasound and CEUS images; the weighted mean of the B-mode ultrasound and CEUS images; the multiplication between the B-mode ultrasound and CEUS images.

**Figure A2:**
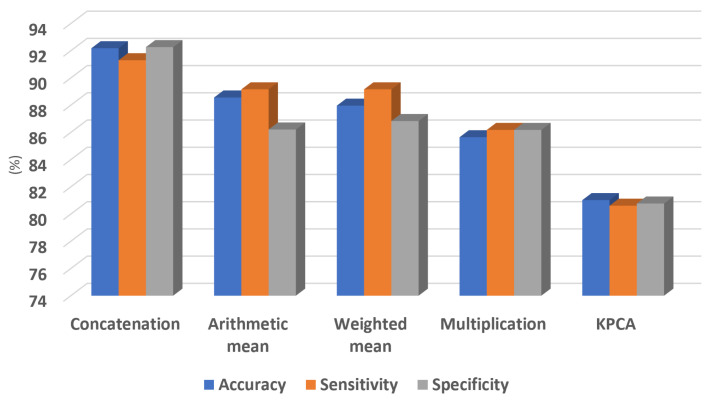
Comparison between the classifier level fusion method.

## Data Availability

The dataset was gathered by the medical specialists from the 3rd Medical Clinic, Iuliu Hatieganu University of Medicine and Pharmacy in Cluj-Napoca, in the context of the project “High accuracy innovative approach for the robotic assisted intraoperatory treatment of hepatic tumours based on imagistic-molecular diagnosis” (IMPROVE), PNIII-P1-1.2-PCCDI2017-0221 Nr.59/1st March 2018.

## Abbreviations

The following abbreviations are used in this manuscript:

ANN	Artificial Neural Networks
AUC	Area Under Curve
BN	Batch Normalization
CT	Computer Tomography
MRI	Magnetic Resonance Imaging
CFS	Correlation-based Feature Selection
CNN	Convolutional Neural Network(s)
DCCA	Deep Canonical Correllation Analysis
DICOM	Digital Imaging and Communications in Medicine
GCM	Generalized Co-occurrence Matrix
GLCM	Gray Level Co-occurrence Matrix
HCC	Hepatocellular Carcinoma
KPCA	Kernel Principal Component Analysis
LDA	Linear Discriminant Analysis
MLP	Multi-Layer Perceptron
PAR	Cirrhotic Parenchyma (on which HCC had evolved)
PCA	Principal Component Analysis
RF	Random Forest
SMO	Sequential Minimal Optimization
SVM	Support Vector Machines

## Appendix A

In Figure A1, relevant examples of HCC and PAR patches are depicted, for both B-mode ultrasound and CEUS image modalities, as well as for the situations when the feature level fusion methods are employed: arithmetic mean, weighted mean, and multiplication, respectively.

## Appendix B

The comparison between the experimented classifier level fusion methods is illustrated in Figure A2, where the arithmetic mean values of accuracy, sensitivity and specificity are illustrated, for each such method. Thus, the best values for these parameters were achieved in the case of concatenation, followed by arithmetic mean and weighted mean that had an almost similar performance, then by multiplication and finally by the KPCA method. The lower performance of KPCA is probably due to the fact that sometimes dimensionality reduction operations had already taken place within the CNN (pooling and dropout operations, respectively 1×1 convolutions).
